# Genetic dissection of maize (*Zea mays*L.) trace element traits using genome-wide association studies

**DOI:** 10.1186/s12870-023-04643-8

**Published:** 2023-12-08

**Authors:** Hang Zhu, Ruiqiang Lai, Weiwei Chen, Chuanli Lu, Zaid Chachar, Siqi Lu, Huanzhang Lin, Lina Fan, Yuanqiang Hu, Yuxing An, Xuhui Li, Xiangbo Zhang, Yongwen Qi

**Affiliations:** 1 Zhongkai University of Agriculture and Engineering, Guangzhou, 510225 Guangdong China; 2 Institute of Nanfan & Seed Industry, Guangdong Academy of Science, Guangzhou, 510316 Guangdong China; 3 Guangdong Laboratory for Lingnan Modern Agriculture, Guangzhou, 510642 Guangdong China; 4 College of Agriculture, Yangtze University, Jingzhou, 434025 Hubei China; 5 Heyuan Provincial Academy of Sciences Research Institute, Guangdong Academy of Sciences, GDAS, Heyuan, 517001 Guangdong China

**Keywords:** Maize, Trace element, GWAS, Crosses combinations, Candidate gene

## Abstract

Maize (*Zea mays* L.) is an important food and feed crop worldwide and serves as a a vital source of biological trace elements, which are important breeding targets. In this study, 170 maize materials were used to detect QTNs related to the content of Mn, Fe and Mo in maize grains through two GWAS models, namely MLM_Q + K and MLM_PCA + K. The results identified 87 (Mn), 205 (Fe), and 310 (Mo) QTNs using both methods in the three environments. Considering comprehensive factors such as co-location across multiple environments, strict significance threshold, and phenotypic value in multiple environments, 8 QTNs related to Mn, 10 QTNs related to Fe, and 26 QTNs related to Mo were used to identify 44 superior alleles. Consequently, three cross combinations with higher Mn element, two combinations with higher Fe element, six combinations with higher Mo element, and two combinations with multiple element (Mn/Fe/Mo) were predicted to yield offspring with higher numbers of superior alleles, thereby increasing the likelihood of enriching the corresponding elements. Additionally, the candidate genes identified 100 kb downstream and upstream the QTNs featured function and pathways related to maize elemental transport and accumulation. These results are expected to facilitate the screening and development of high-quality maize varieties enriched with trace elements, establish an important theoretical foundation for molecular marker assisted breeding and contribute to a better understanding of the regulatory network governing trace elements in maize.

**Supplementary Information:**

The online version contains supplementary material available at 10.1186/s12870-023-04643-8.

## Introduction

Maize (*Zea mays* L.), recognized as a vital staple crop for both human consumption and animal feed, is known as a “longevity food” because of its rich nutrient content comprising essential vitamins, protein and beneficial trace elements [1]. While research into trace elements was initiated as early as 1925 [2], a significant number of people suffer from Fe and Zn deficiencies, particularly among populations heavily reliant on cereals as their primary staple food [3–5]. Trace elements are mineral substances that exist in minute quantities within living tissues and are crucial for a multitude of metabolic, and developmental processes. Iron (Fe), zinc (Zn), manganese (Mn), iodine (I), copper (Cu), selenium (Se), molybdenum (Mo), and cobalt (Co) are indispensable essential trace elements that function as cofactors in a wide range of enzymatic reactions. These trace elements are of utmost importance and have significant impacts on overall human well-being, affecting various aspects of physical and cognitive health [6]. Understanding the genetic determinants of trace element accumulation in crops such as maize is pivotal for enhancing the nutritional quality of food and addressing global health challenges. Trace elements, despite their name, play a crucial role in both plant and human nutrition. Iron (Fe), manganese (Mn), copper (Cu), and zinc (Zn) are essential micronutrients required for various biological processes. However, elements like cadmium (Cd) and arsenic (As) are toxic even at low concentrations, posing a risk to health when they enter the food chain [7].

Furthermore, various elements, are including toxic elements such as cadmium, are assimilated from the soil by roots to the seed. Therefore, finding quantitative trait loci (QTL) related to these elements and breeding varieties that are enriched with beneficial elements without toxic elements is an important means to improve the overall quality of human life.

Currently, numerous studies have been conducted to identify QTLs and candidate genes involved in the production of different elements in plants. In particular, the *MOT1* gene responsible for molybdenum (Mo) [8, 9], the *HMA3* gene associated with cadmium (Cd) [10], the *HAC1* gene linked to arsenic (As) [11] have been successfully identified using GWAS in *Arabidopsis*. Similarly, in rice, previous researchers have determined a significant number of QTLs for specific elements using bi-parental populations and association mapping panels [12–18]. Furthermore, some candidate genes have been accurately mapped, such as *OsHMA3* for Cd [19, 20], and *OsHMA4* for copper (Cu) [21]. In maize, several QTLs and candidate genes associated with different elements have been identified through linkage and association mapping [22–27]. Although these studieshave made noteworthy progress in identifying stable QTLs or quantitative trait nucleotides (QTNs) for different elements, the number of excavations is limited, and no researchers have used them to evaluate element accumulation in maize material. There is a need to expand the range of research to detect more loci for breeding improvement in most staple crops, including maize.

Maize, is one of the top three major food crops in the world, especially the largest cultivated crop grown in China, and it is important to map novel and stable loci that control the accumulation of different elements to achieve high-quality maize cultivation. However, although previous studies have mostly used a limited number of molecular markers, such as SSR or InDel, to probe loci associated with target traits, compared with SNP, these traditional methods with less marker density and lower localization accuracy have limited efficiency. Currently, an efficient GWAS method utilizes more recombination events and abundant SNP markers. This methodology enables a higher resolution and facilitates the discovery of more comprehensive genetic information regarding traits of interest. For example, using SNP markers, 23 and 38 QTNs related to shoot and blossom blight resistance, respectively were detected in 273 apple accessions [28]. Similarly, 18 QTNs have been found to be associated with stem rot resistance in soybean [29]. Unfortunately, GWAS areseldom featured in the genetic dissection of maize elemental traits.

Additionally, soil in many parts of the world lacks trace elements. For example, approximately 30% of the soils in China are deficient in manganese (Mn) and (Mo) molybdenum, while 40% lack iron (Fe) [30]. These deficiencies negatively affect the growth and development of plants, thereby hindering the cultivation of high yield and high-quality maize. To address this issue, we conducted genotyping-by-sequencing (GBS) of 170 maize accessions from a natural-variation germplasm pool. This allowed us to identify an abundant of SNPs for explaining the genetic basic of Mn, Fe, and Mo elements in maize grain. Our objectives were to (1) detect peak SNPs and their superior alleles related to element traits using GWAS, (2) predict the best cross combination using superior alleles, (3) identify potential candidate genes responsible for elemental traits based on peak SNPs.

## Results

### Phenotypic evaluation for trace elements in an association panel

**Fig. 1:**
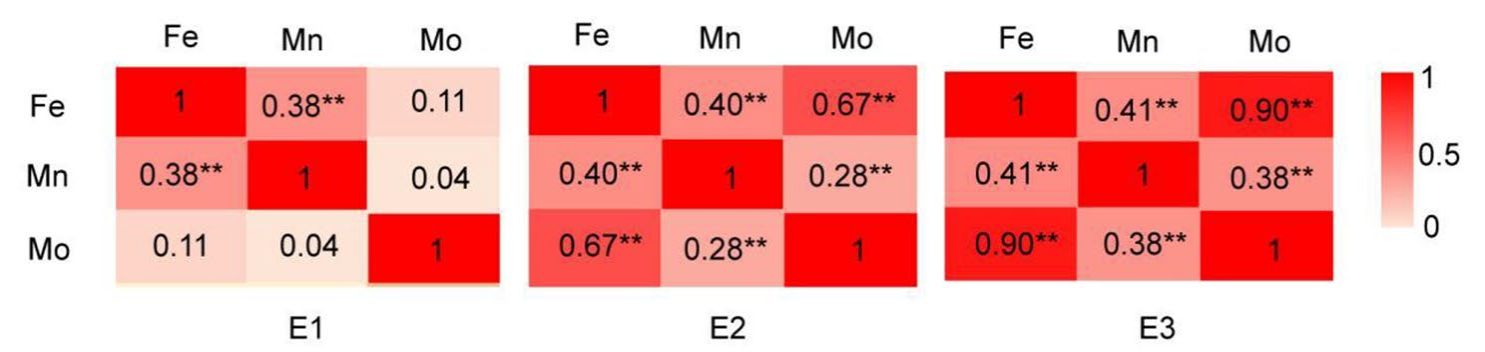
The correlations analysis among Fe, Mn, and Mo element in three environments. E1: Hainan experimental station (2013); E2: Jiangmen experimental station (2020); E3: Jiangmen experimental station (2021); The number in the rectangle is the correlation coefficient, the closer to “1”, the closer red; **: significance at *P*-value < 0.01

The statistical analysis aimed to assess elemental traits in different environments, especially Mn, Fe and Mo (Table 1). The mean, range, standard deviation, skewness, kurtosis, coefficient of variation (CV), and broad-sense heritability were calculated to evaluate these traits. The CV for Mo exceeded 50% in all environments, while the CV range for Fe was 21.77 ~ 95.38%, and for Mn it was 32.84 ~ 35.75%. These results indicate the presence of phenotypic variation in element traits among the accessions (Table 1). Analysis of gene and environmental effects (Table 1) revealed that both factors significantly influenced elemental traits. Furthermore, broad-sense heritability (Table 1) ranged from 71.54 ~ 79.92%, indicating the genetic factors still play an important role. Correlation analysis (Fig. 1) showed a significant correlation between Fe and Mn elements in all three environments. However, no significant correlation was found between Mo and Fe or Mn in E1, but Mo was found to be significantly correlated with Fe and Mn in both E2 and E3, indicating that the environment has a greater impact on the relationship between Mo accumulation and Mn accumulation, as well as between Mo and Fe than between Mn and Fe, respectively.

**Table 1:** Descriptive statistics for ionomic elements in 170 maize accessions in three environments

Elements	Environment	Mean	Rang	SD	CV/%	Kurtosis	Skewness	Fg	Fenv	Fg×env	h^2^B/%
Mn	E1	9.78	0 ~ 22.81	3.29	33.59	1.70	0.77	23.01**	884.15**	12.03**	77.95
	E2	7.02	1.30 ~ 14.64	2.50	35.62	0.45	0.64				
	E3	7.54	1.20 ~ 15.96	2.47	32.72	1.12	0.87				
Fe	E1	29.59	9.61 ~ 46.58	6.14	20.76	0.07	0.06	3.10**	110.83**	1.47*	71.54
	E2	49.38	0 ~ 269.89	46.93	95.04	3.27	1.38				
	E3	77.24	0 ~ 357.80	50.91	65.91	7.72	2.12				
Mo	E1	0.21	0 ~ 0.73	0.13	60.05	1.57	1.22	42.80**	5512.91**	20.50**	79.92
	E2	1.08	0.30 ~ 3.85	0.63	58.41	4.19	1.69				
	E3	1.20	0.06 ~ 4.12	0.70	58.55	4.28	1.71				

E1: Hainan experimental station (2013); E2: Jiangmen experimental station (2020); E3: Jiangmen experimental station (2021); SD: standard deviation; CV: coefficient of variation; Fg, Fenv, F_g×env_: F values in ANOVA for genotype, environment, genotype × environment, respectively; *, **: significance at *P*-value < 0.05, 0.01, respectively; h^2^B: broad-sense heritability

### Genome-wide association mapping for elements

**Fig. 2:**
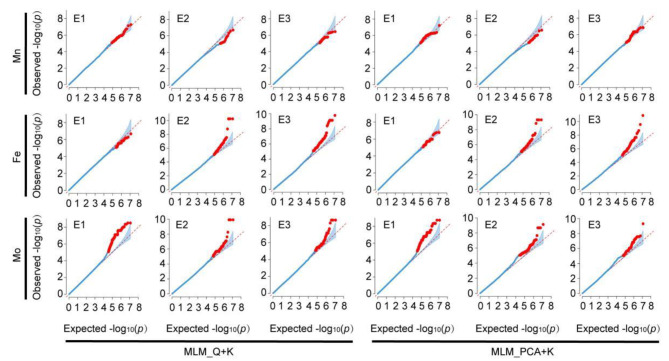
Two GWAS models for the control of false positive (Q-Q plots). The X-axis and Y-axis is expected -log_10_(*p*) and observed -log_10_(*p*) of the Mn, Fe, and Mo element concentration in maize grain, respectively; The Q-Q plots of two models include MLM_Q + K on the left and MLM_PCA + K on the right; E1: Hainan experimental station (2013); E2: Jiangmen experimental station (2020); E3: Jiangmen experimental station (2021)

**Fig. 3:**
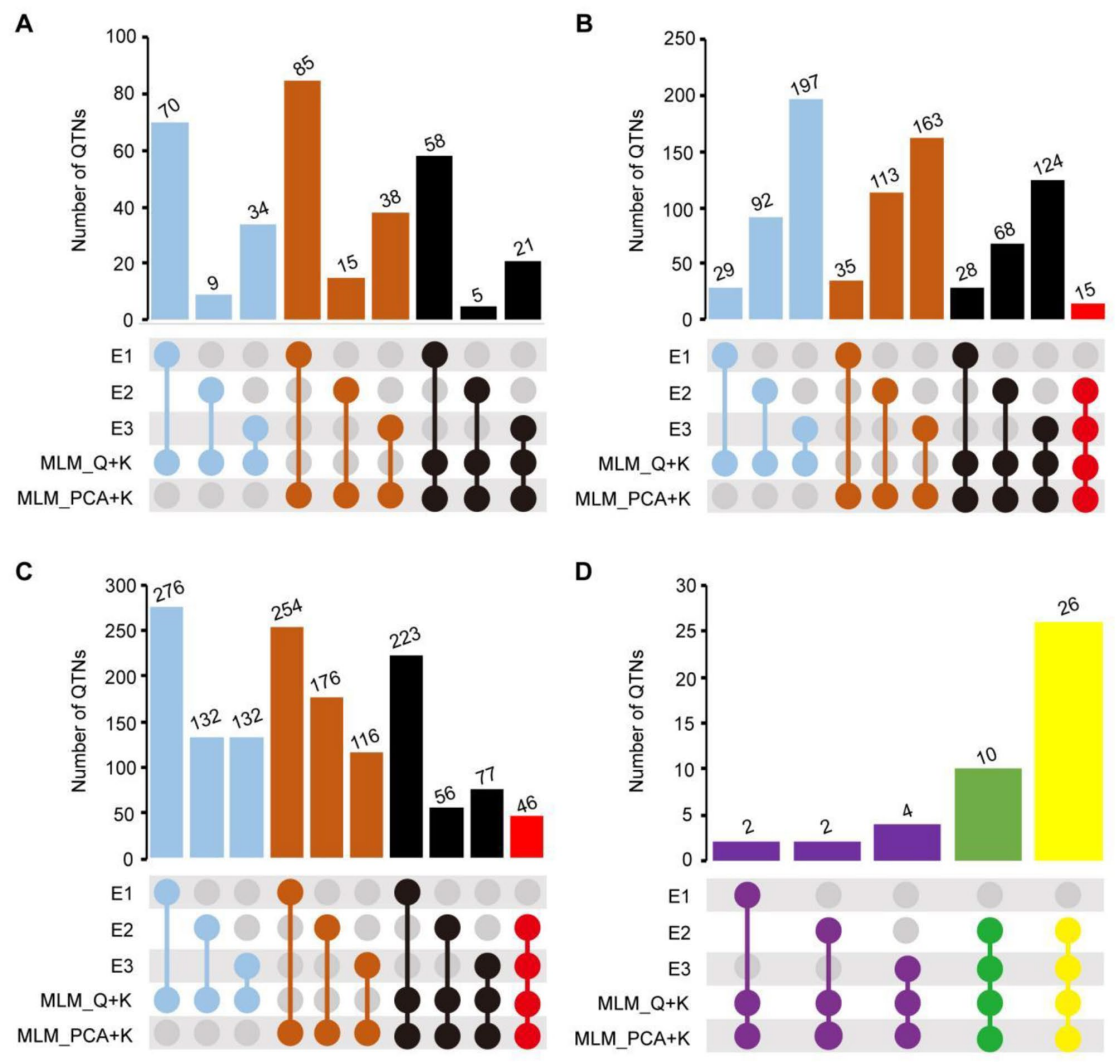
The number of significant QTNs and stable QTNs for the concentration of Mn, Fe, and Mo element identified in three environments and GWAS models. **(A)** Mn element; **(B)** Fe element; **(C)** Mo element; the color with blue when the model used MLM_Q + K in single environment, but the color with brown mean the model using MLM_PCA + K in single environment; and the black color mean two model was used in single environment; red color was used that mean QTNs which was found not only using two models, but also found in two environment. **(D)** Number of Stable QTLs of Mn element (purple), Fe element (green), and Mo element (yellow). E1: Hainan experimental station (2013); E2: Jiangmen experimental station (2020); E3: Jiangmen experimental station (2021). Horizontal bars show the number of QTNs for different environments and methods. The colors of circles corresponding to Horizontal bars indicate the environment in which QTNs was detected and the method applied

Population structure and principal component analysis were performed to obtain the.

correction coefficients, Q and PCA, respectively. Moreover, the kinship coefficient K was used as a correction coefficient. Subsequently, two GWAS methods (Fig. 2), suitable for the data from this study, including the MLM_ Q + K model and MLM_ PCA + K models, were used to detect QTNs. The results show that the MLM_Q + K model detected significant associations of 70 (E1), 9 (E2) and 34 (E3) SNPs with Mn (*P* < 4.31E-6). Similarly, the model found significant associations of 29 (E1), 92 (E2) and 197 (E3) SNPs with Fe, and 276 (E1), 132 (E2) and 132 (E3) SNPs with Mo (Fig. 3A and B C; Table S1). Using the MLM_Q + PCA model, 85/15/38 significant SNPs associated with Mn, 35/113/163 with Fe, and 254/176/116 with Mo were detected in environmental context E1/E2/E3, respectively (Fig. 3A and B C; Table S1).

Moreover, 87 significant SNPs (Fig. 3A; Table S2) detected by both methods were associated with Mn element in three environments. These SNPs were located on Chr1 ~ Chr4 and Chr7 ~ Chr10, with a *P*-value distribution ranging from 3.51E-06 ~ 6.16E-08. Similarly, 205 SNPs (Fig. 3B; Table S2) related to Fe element in three environments were found on Chr1 ~ Chr10, and the *P*-value distribution ranging from 4.11E-06 ~ 1.32E-11. Additionally, 310 SNPs (Fig. 3C; Table S2) related to Mo element in three environments were identified on Chr1 ~ Chr10, and the *P*-value distribution ranging from 4.09E-06 ~ 4.95E-10.

### Identification of reliable quantitative trait nucleotides

Significant SNPs detected through two different methods and across multiple environments were regarded as reliable QTNs., Our analysis identified 15 SNPs related to Fe element (Fig. 3B; Table S3) in two GWAS models. The corresponding *P*-value ranged from 4.07E-06 ~ 4.87E-10, and they were detected in E2 and E3. Similarly, 46 SNPs related to Mo element (Fig. 3C; Table S3) were detected in E2 and E3, and the *P*-value distribution was 4.14E-06 ~ 7.38E-10. Interestingly, we observed the presence of 10 SNPs (Table S3) that are significantly related to both Fe and Mo elements. These SNPs were categorized as multiple effect SNPs and were located on Chr4 (*qFe/Mo-4*), Chr5 (*qFe/Mo-5*), Chr7 (*qFe/Mo-7*), Chr8 (*qFe/Mo-8a*; *qFe/Mo-8b*; *qFe/Mo-8c*), Chr9 (*qFe/Mo-9*), and Chr10 (*qFe/Mo-10a*; *qFe/Mo-10b*; *qFe/Mo-10c*).

In addition, although Mn element related SNPs were not co-located in multiple environments, we aimed to enhance the selection of high-quality candidate materials related to trace elements. To achieve this, we applied a stricter screening threshold of Mn related SNPs by considering only those with a *P*-value smaller than 0.05/116,011 = 4.31E-7 as more reliable peaks. From this, 8 SNPs (Fig. 3D; Table S3) were associated with Mn element, located on Chr1 (*qMn-1a*; *qMn-1b*; *qMn-1c*) Chr3 (*qMn-3a*; *qMn-3b*; *qMn-3c*), Chr4 (*qMn-4*) and Chr7 (*qMn-7*).

### Identification of stable QTNs and superior alleles for ionomic elements

The detected of QTNs during breeding is the goal of molecular marker assisted breeding. In this research, the evaluation of superior alleles in reliable QTNs in different environments was undertaken using a cohort of 130 materials with 0 phenotypic deletion rate in three environments. These materials served as the basis for the evaluation and subsequent selection. Subsequently, 8, 15 and 46 SNPs related to Mn, Fe, and Mo element, respectively, were identified. Here, the phenotypic effect value was calculated for QTN considered to be reliable, in order to understand the effect of a single QTN on the corresponding phenotype.

**Fig. 4:**
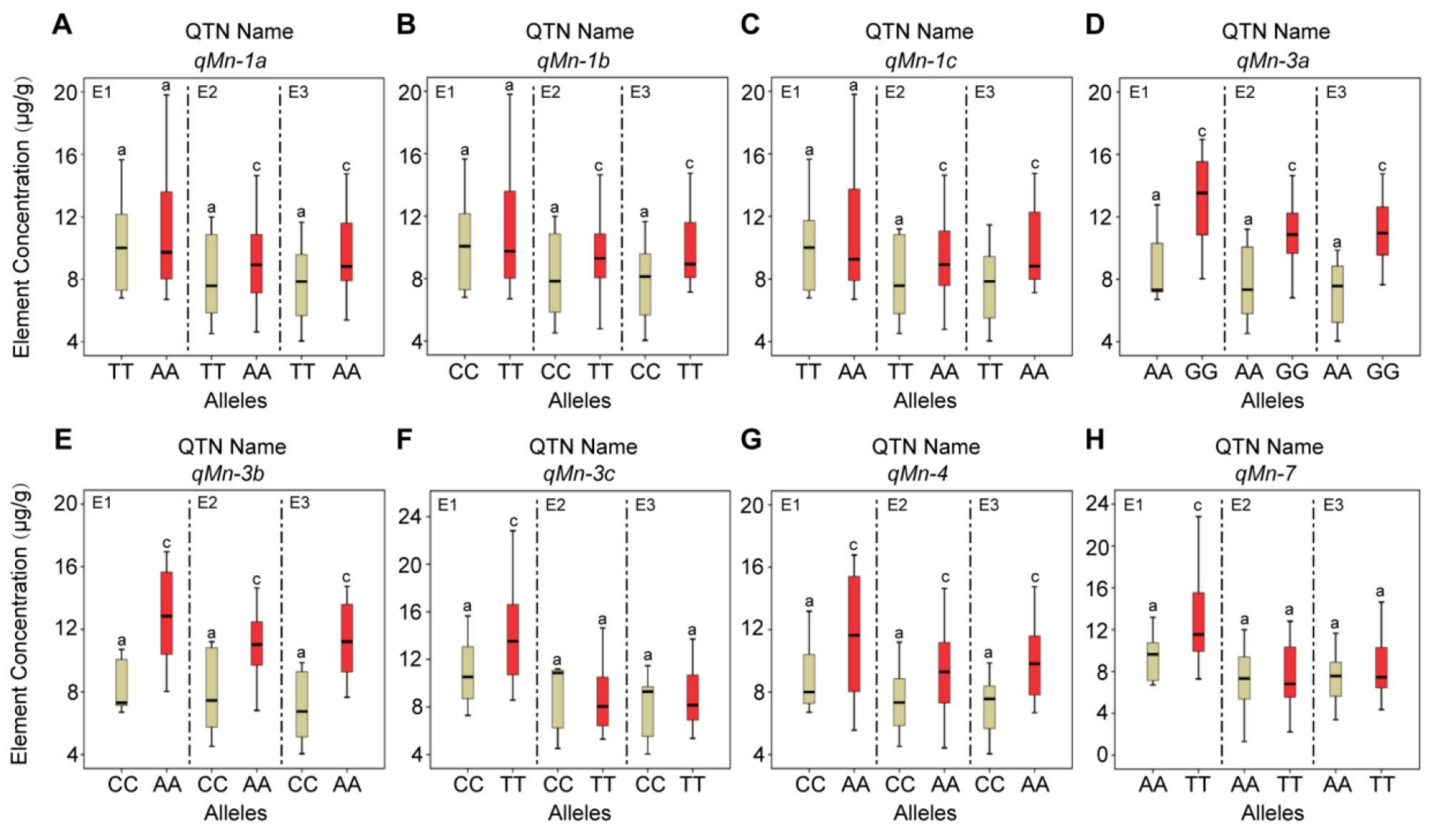
The concentration of Mn element differences between superior and alternative alleles of each QTN of maize in different environments. **(A-H)**: Alleles corresponding to red box-plot are superior alleles, while brown box-plot are alternative alleles. E1: Hainan experimental station (2013); E2: Jiangmen experimental station (2020,); E3: Jiangmen experimental station (2021). Different letters indicate significant differences, with significance at P-value < 0.01 (a and c) using ANOVA.

8 QTNs (Figs. 3D and 4A-H; Table S4) related to Mn element showed a positive trend in the accumulation of Mn in three environments, indicating their stability. The synergistic level of superior alleles was 0.5550 ~ 4.3721 µg/g, while the alternative allele showed reduction ranging from − 0.5777~-0.0770 µg/g. The accumulation of Mn among materials with different alleles of *qMn-1a* (Fig. 4A), *qMn-1b* (Fig. 4B) and *qMn-1c* (Fig. 4C) showed significant differences (*P* < 0.01) in environmental E2 and E3, and the accumulation of Mn among materials with different alleles of *qMn-3c* (Fig. 4F) and *qMn-7* (Fig. 4H) showed significant differences only in environmental E1. Excitingly, the superior alleles GG, AA, and AA of *qMn-3a* (GG), *qMn-3b* (AA), and *qMn-4* (AA) revealed a significant positive effect on Mn accumulation across all three environments, with synergistic levels ranging from 3.2689 ~ 3.8682 µg/g, 3.1708 ~ 3.9973 µg/g and 1.7293 ~ 2.2727 µg/g, respectively. While the alternative allele of AA, CC and CC exhibited a negative effect with a reduction level ranging from − 0.3576 ~ -0.3022 µg/g, -0.3331 ~ -0.2642 µg/g and − 0.3791~-0.2602 µg/g, respectively (Fig. 4D, E and G; Table S4).

**Fig. 5:**
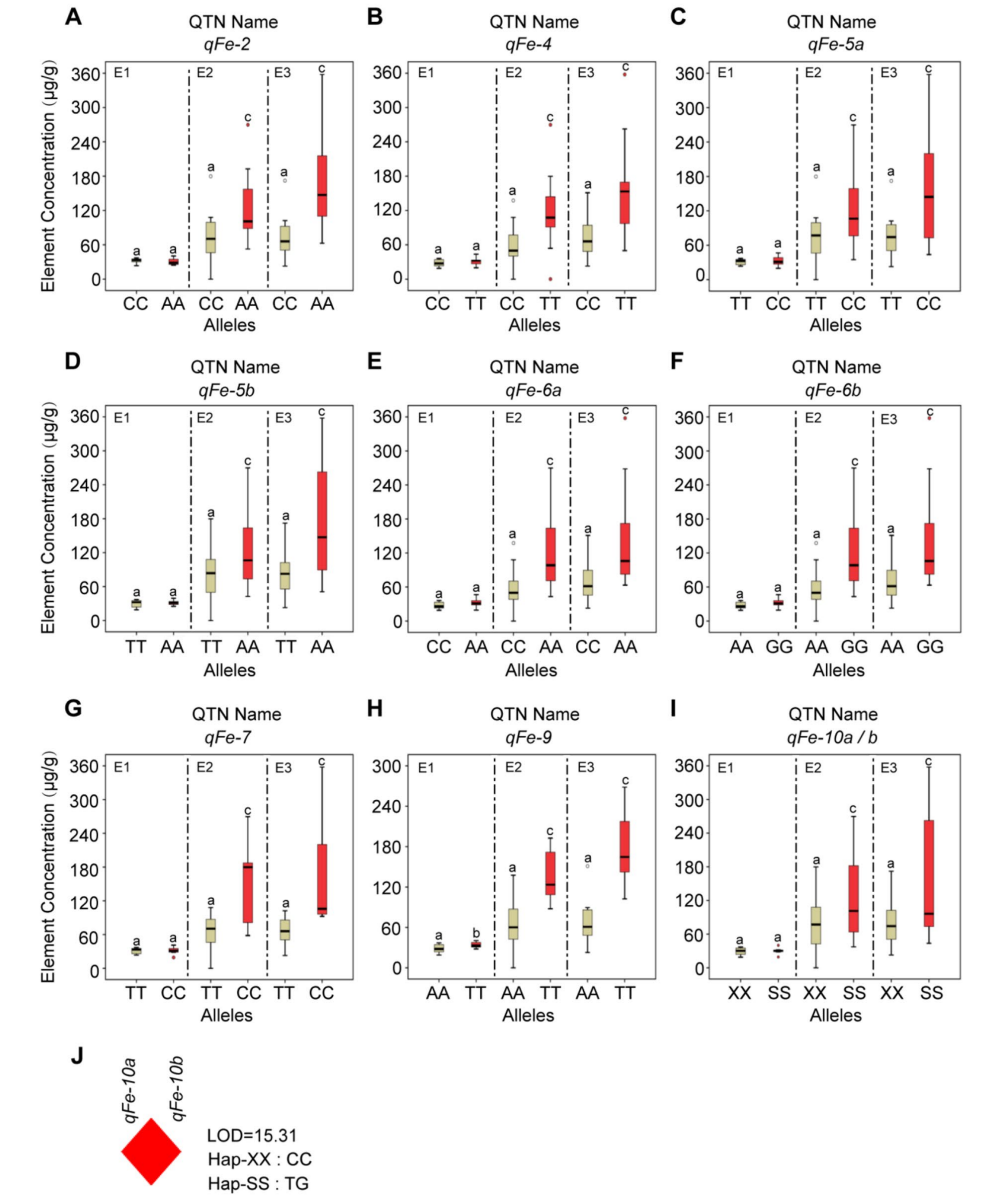
The concentration of Fe element differences between superior and alternative alleles of each QTN of maize in different environments. **(A-I)**: Alleles corresponding to red box-plot are superior alleles, while brown box-plot are alternative alleles. E1: Hainan experimental station (2013); E2: Jiangmen experimental station (2020); E3: Jiangmen experimental station (2021). Different letters indicate significant differences, with significance at P-value < 0.05 (a and b), 0.01 (a and c) using ANOVA. XX represents alternative alleles CC and CC for *qFe-10a* and *qFe-10b*, respectively; SS represents superior alleles TT and GG for *qFe-10a* and *qFe-10b*, respectively. **(J)**: Analysis of linkage disequilibrium; red-diamond represents they are complete linkage (r^2^ = 1), and the Hap-XX and Hap-SS corresponds to the type of the haplotype

10 out of 15 QTNs related to Fe element (Figs. 3D and 5A-I; Table S3, S4) showed a positive trend in the accumulation of Fe in three environments. These QTNs were considered stable, and the synergistic level of superior alleles was 0.4295 ~ 105.8084 µg/g, whereas the reduction level of alternative allele ranged from − 8.0681~-0.0399 µg/g. Among them, only *qFe-9* (Fig. 5H) reached a significant level (*P*-vlaue < 0.05) in all three environments, the corresponding superior allele was TT, and the synergistic level was 4.1660 ~ 98.5975 µg/g. Conversely, the alternative allele (AA), resulted in a reduction ranging from − 6.4654~-0.2187 µg/g. However, the remaining nine QTNs (Fig. 5A-G and I) reached significant levels in environments E2 and E3, but not in E1. For instance, the QTN *qFe-2* (Fig. 5A; Table S4), showed statistical significance, with the superior allele being AA, and the synergistic level ranging from 0.8746 ~ 95.2883 µg/g. In contrast, the alternative allele CC led to a reduction ranging from − 5.4229~-0.0498 µg/g in the three environments. Furthermore, we observed the same QTN effects for *qFe-10a* and *qFe-10b* (Fig. 5I; Table S4). Given the analysis of linkage disequilibrium, *qFe-10a* and *qFe-10b* are in complete linkage (r^2^ = 1) (Fig. 5J).

**Fig. 6:**
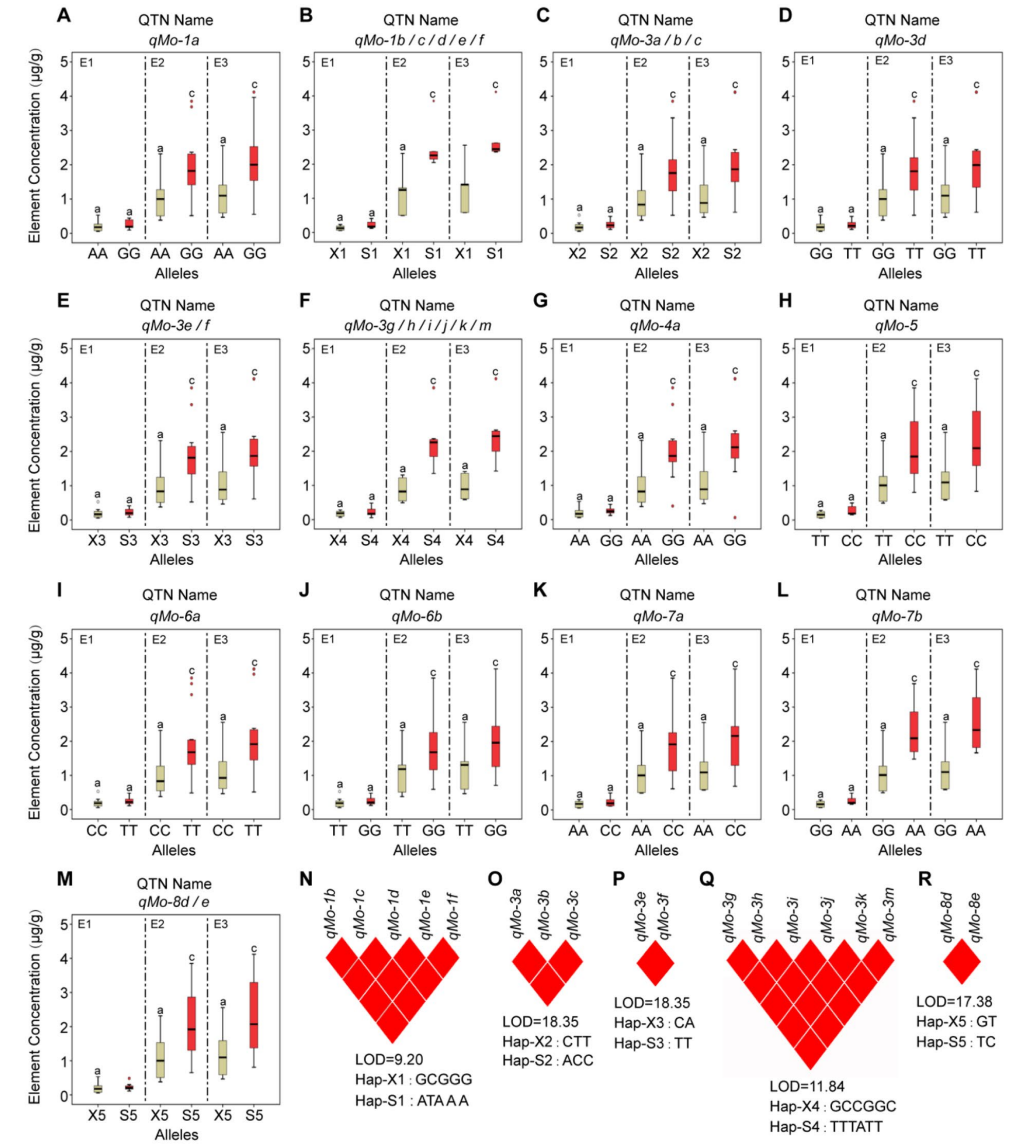
The concentration of Mo element differences between superior and alternative alleles of each QTN of maize in different environments. **(A-M)**: Alleles corresponding to red box-plot are superior alleles, while brown box-plot are alternative alleles. E1: Hainan experimental station (2013); E2: Jiangmen experimental station (2020); E3: Jiangmen experimental station (2021). Different letters indicate significant differences, with significance at P-value < 0.01 (a and c) using ANOVA. X1 represents alternative alleles GG, CC, GG, GG, and GG for *qMo-1b*, *qMo-1c*, *qMo-1d*, *qMo-1e*, and *qMo-1f*, respectively; S1 represents superior alleles AA, TT, AA, AA, and AA for *qMo-1b*, *qMo-1c*, *qMo-1d*, *qMo-1e*, and *qMo-1f*, respectively. X2 represents alternative alleles CC, TT, and TT for *qMo-3a*, *qMo-3b*, and *qMo-3c*, respectively; S2 represents superior alleles AA, CC, and CC for *qMo-3a*, *qMo-3b*, and *qMo-3c*, respectively. X3 represents alternative alleles CC, and AA for *qMo-3e*, and *qMo-3f*, respectively; S3 represents superior alleles TT, and TT for *qMo-3e*, and *qMo-3f*, respectively. X4 represents alternative alleles GG, CC, CC, GG, GG, and CC for *qMo-3 g*, *qMo-3 h*, *qMo-3i*, *qMo-3j*, *qMo-3k*, and *qMo-3 m*, respectively; S4 represents superior alleles TT, TT, TT, AA, TT, and TT for *qMo-3 g*, *qMo-3 h*, *qMo-3i*, *qMo-3j*, *qMo-3k*, and *qMo-3 m*, respectively. X5 represents alternative alleles GG, and TT for *qMo-8d*, and *qMo-8e*, respectively; S5 represents superior alleles TT, and CC for *qMo-8d*, and *qMo-8e*, respectively. **(N-R)**: Analysis of linkage disequilibrium; red-diamond represents they are complete linkage (r^2^ = 1), and the Hap-X1 ~ X5 and Hap-S1 ~ S5 corresponds to the type of the haplotype

Similarly, 26 of the 46 QTNs related to Mo element (Figs. 3D and 6A-M; Table S3, S4) showed a positive trend in the accumulation of Mo elements in the three environments, therefore they were considered to stable QTNs, and their synergistic level of superior alleles was 0.0108 ~ 1.5583 µg/g. The reduction level of alternative alleles ranged from − 0.1236~-0.0007 µg/g. All these QTNs reached a significant level (*P* < 0.01) in E2 and E3, but not in E1 (Fig. 6A-M). For example, the superior allele of *qMo-5* is CC, with a synergistic level ranging from 0.0544 to 1.1079 µg/g, while the reduction level of TT allele is -0.0036~-0.0726 µg/g in three environments (Fig. 6H; Table S4). It is worth mentioning that *qMo-5* and *qFe-5a* belong to the same QTN (*qFe/Mo-5*), and both superior alleles are CC (Figs. 5C and 6H; Table S4). They are relatively stable and reliable multi effect QTN, which can be focused on the subsequent material screening. Furthermore, five groups of QTNs revealing similar effects, including *qMo-1b*, *qMo-1c*, *qMo-1d*, *qMo-1e* and *qMo-1f* (Fig. 6B; Table S4); *qMo-3a*, *qMo-3b* and *qMo-3c* (Fig. 6C; Table S4); *qMo-3e* and *qMo-3f* (Fig. 6E; Table S4); *qMo-3 g*, *qMo-3 h*, *qMo-3i*, *qMo-3j*, *qMo-3k* and *qMo-3 m* (Fig. 6F; Table S4); *qMo-8d* and *qMo-8e* (Fig. 6M; Table S4). Given the analysis of linkage disequilibrium, QTNs are completely linked (r^2^ = 1) in the same group (Fig. 6N-R).

### Distribution of superior alleles and prediction of cross combination

Seven (5.38%) ~ 23 (17.69%) materials including superior alleles of each of the eight stable QTNs associated with Mn element. Conversely, the majority of the material, 107 (82.31%) ~ 123 (94.62%), exhibited alternative alleles of each of these eight QTN (unfavorable for element accumulation). Among these, 20 materials containing 1 (15%) ~ 7 (5%) superior alleles have a high accumulation of Mn in three environments (Table S5), of whichCAU425 (15.0427 µg/g, mean of element content in three environments) has seven superior alleles. In contrast, CAU115 (12.1150 µg/g), CAU119 (12.6853 µg/g), and IL59 (11.6607 µg/g) have four superior alleles each, with one unique superior allele exclusive to each of the latter three materials but absent in CAU425 (Table 2, S5).

**Table 2:** Best parental cross combinations for trace elements in maize from superior alleles

Direction	P1	P2	P1- Phenotypt# (µg/g)	P2- Phenotypt# (µg/g)	P1-Superior Alleles	P1-Superior Alleles	Expected offspring Alleles
**Higher Mn**	CAU425	CAU115	15.0427	12.1150	7	4	8
	CAU425	CAU119	15.0427	12.6853	7	4	8
	CAU425	IL59	15.0427	11.6607	7	4	8
**Higher Fe**	CAU268	CAU252	164.0771	152.2168	9	7	10
	CAU268	CAU106	164.0771	128.0680	9	5	10
**Higher Mo**	CAU254	CAU78	1.6391	1.6018	13	12	24
	CAU252	CAU78	2.5735	1.6018	12	12	20
	CAU254	CAU342	1.6391	1.5765	13	11	19
	CAU78	CAU342	1.6018	1.5765	12	11	19
	CAU254	CAU252	1.6391	2.5735	13	12	18
	CAU252	CAU342	2.5735	1.5765	12	11	18
**Higher Mn/Fe/Mo**	CAU425	CAU78	15.0427/103.2969 /1.6063	9.3486/114.4238/1.6018	7/3/21	0/3/12	7/6/26
	CAU425	CAU252	15.0427/103.2969 /1.6063	9.7351/152.2168/2.5735	7/3/21	4/7/12	7/8/25

#: Phenotypic values are the mean of element concentrations in three environments

Seven (5.38%) ~ 13 (10%) materials have superior alleles of the 10 stable QTNs associated with Fe element, and 117 (90%) ~ 123 (94.62%) materials have alternative alleles. Among these, 19 materials containing 1 (42.11%) ~ 9 (5.26%) superior alleles have higher Fe accumulation in three environments (Table S5). Material CAU268 (164.0771 µg/g) has 9 superior alleles and material CAU252 (152.2168 µg/g) and CAU106 (128.0680 µg/g) has 7 and 5 superior alleles, respectively, and they feature another superior allele that CAU268 does not have (Table 2, S5).

Five (3.85%) ~ 16 (12.31%) materials have superior alleles of the 26 stable QTNs associated with Mo element, and 114 (87.69%) ~ 125 (96.15%) materials have alternative alleles. Among these, 17 materials containing 1 (11.76%) ~ 13 (5.88%) superior alleles were identified to have higher Mo accumulation in three environments (Table S5). CAU254 (1.6391 µg/g), CAU78 (1.6018 µg/g), CAU252(2.5735 µg/g), and CAU342 (1.5765 µg/g) has 13, 12, 12, and 11 superior alleles, respectively, including 26 QTNs (Table 2, S5) which can be used as candidate materials for optimizing Mo element accumulation.

To obtain materials with high levels of trace elements, several cross combinations of materials can be leveraged to select offspring (Table 2). Three hybrid combinations, namely CAU425 × CAU115、CAU425 × CAU119 and CAU425 × IL59, have the potential to increase Mn accumulation in derived hybrids. Two hybrid combinations, namely, CAU268 × CAU252 and CAU268 × CAU106, could significantly improve Fe accumulation in hybrid materials. To obtain Mo materials with a high accumulation a combination of among CAU254, CAU78, CAU252, and CAU342 are promising. For instance, the hybrids derived from CAU254 × CAU78 are expected to contain 24 superior alleles.

In addition, CAU425 has a high accumulation of Mn and contains the most relevant superior alleles. While the CAU78 displayed high accumulation of Fe (114.4238 µg/g) and Mo, with 3 and 12 superior alleles, respectively and contains *qFe/Mo-5* (Table S5). Moreover, the Fe and Mo accumulation level of CAU252 in different environments is high (Table S5), which can also be used as a candidate material. Therefore, the cross combinations CAU425 × CAU78 or CAU425 × CAU252 can be utilized to breed high-quality offspring with high accumulation of Mn/Fe/Mo (Table 2).

### Identification of candidate genes base on SNP peak

Here, we present information on candidate genes associated with each QTNs based on the ± 100kbp regions that include three elements (Table S6). We specifically focused on the candidate genes related to Mn, Fe and Mo elements, identified 8, 10 and 26 QTNs, respectively. Through mining efforts, we discovered 22 (Mn), 32 (Fe) and 49 (Mo) candidate genes (Table S3, S6), and the corresponding protein IDs were obtained (Table S7). GO analysis was conducted on each of the candidate genes (adjust *P*-value < 0.05), and three candidate genes (Table 3, S7, S8) related with Mn belonged to 5 biological processes (BP), 2 molecular function (MF), and 1 cellular component (CC). For example, Zm00001eb028460 was located 84.926kbp downstream *qMn-1c* (Table S3, S6). Two candidate genes (Table 3, S7, S8) related to Fe were found to involve 2 BPs, including ribosomal large subunit biology (GO: 0042273) and mRNA translation (GO: 0009299). Seven candidate genes (Table 3, S7, S8) related to Mo belonged to 2 PB, 5 MF, and 3 CC. For example, Zm00001d034856 was located 1.24kbp upstream *qMo-1a* (Table S3, S6), expressing a putative translation elongation/initialization factor family protein involved in the mitochondrial translation elongation (GO: 0070125; Table 3, S8) and has a translation elongation factor activity (GO: 0003746, Table 3, S8). In addition, KEGG analysis (adjust *P*-value < 0.05) found that 1 candidate gene involved 1 KEGG pathway (zma00960), which could be associated with the accumulation of Mn element (Table 3, S7, S8).

**Table 3:** The GO and KEGG information of candidate genes

Related element	Genes ID	Gene Ontology ID			KEGG ID
		BP	MF	CC	
**Mn**	Zm00001eb028460	GO:0030970/GO:0030433	-	GO:0005788	-
	Zm00001d042152	GO:0006072	GO:0004367	-	-
	Zm00001eb028430	GO:0045893	GO:0000976	-	-
	Zm00001d048723	-	-	-	zma00960
**Fe**	Zm00001d025857	GO:0042273	-	-	-
	Zm00001d038016	GO:0009299	-	-	-
**Mo**	Zm00001d038903	-	GO:0004045	-	-
	Zm00001d034855	-	-	GO:0042788/GO:0005854	-
	Zm00001d034856	GO:0070125/GO:0006414	GO:0003746	-	-
	Zm00001d039674	-	-	GO:0005794	-
	Zm00001d038676	-	-	GO:0005794	-
	Zm00001d038904	-	GO:0030170	-	-
	Zm00001d010476		GO:0008353/GO:0004693		-

- means there is no corresponding information

## Discussion

Maize is an important economically crop and act as a model plant for genetic studies [24]. There are several reasons for focusing on the trace elements in maize grains. First, maize grains more important in the production process than other parts, and trace elements in grains are closely related to grain quality and geographical adaptation. Secondly, the accumulation of trace elements in plants is an important factor that affects growth and development, and ultimately yield, falling under the umbrella of quantitative genetics.

In our study, phenotypic data analysis showed that Mn, Fe and Mo elements in maize grains were greatly affected by environment, yet also being significantly affected by genetic factors. This finding indicates that it is feasible to manipulate the content of trace elements in maize grains, paving the way to the development of maize varieties with high quality, high yield and extensive geographical adaptability.

Population structure can lead to false correlation in association analyses. For example, Flint-Garcia et al. [31] found that the average phenotypic variation explained by population stratification was about 9.3%, resulting in false associations with loci caused by non-functional genes. In addition,, it is beneficial to weaken the false correlation by adding a kinship coefficient in an association analysis [32]. Moreover, earlier research suggested that QTNs identified by multiple methods are similar to environmentally stable QTNs and are reliable [33]. In our study, we employed population structure analysis and principal component analysis to reduce false associations caused by population stratification using the MLM_Q + K and MLM_ PAC + K models for GWAS. Through these approaches, we successfully identified multiple QTNs associated with Mn, Fe, Mo elements in three environments, and searched for candidate gene information near these QTNs, laying the foundation for future research.

To identify more stable and high-quality candidate materials, this study employed a scientific approach by combining various environmental factors, screening thresholds, and phenotypic values across three different environments. We identified a set of stable QTNs related to Mn (8), Fe (10) and Mo (26) and identified their corresponding superior alleles. This information facilitates the breeding of maize varieties with high Mn, Fe, and Mo contents through molecular breeding. We also used these superior alleles to predict the best cross combinations for producing maize hybrids with high concentration of trace elements in the three environments, following the single parent participated in multiple crosses approach described in previous studies [34–36]. For instance, Jiayang Li’s team leveraged on superior alleles developed a high-yielding rice variety LYP9 through molecular breeding [34, 37]. These new superior alleles will improve our ability to enrich trace elements in maize and optimize their quality.

We also examined CAU425, which holds superior alleles related to Mo than the recommended candidates (CAU254, CAU78, CAU252, and CAU342). Since CAU425 did not show high accumulation Mo at E1, suggesting a relative instability, it was not selected as a preferable candidate. Further verification across multiple environments would provide more reliable locus information.

The detection of most micro-effect genes is challenging due to their low single effect ability, making it even more difficult to locate stable QTNs in various environments. Here, we integrated multiple factors, focusing on the candidate genes near eight QTNs related to Mn, ten QTNs related to Fe, and twenty-six QTNs related to Mo. Through genome comparison, literature search, GO and KEGG analyses, we identified 13 candidate genes that may directly or indirectly participate in the transport process of Mn (4), Mo (7), and Fe (2), of which, 10 have homologous genes in *Arabidopsis thaliana*. For example, the homologous gene AT5G35080 of Zm00001eb028460 encodes a glycoprotein located in the endoplasmic reticulum, involved in the process of endoplasmic reticulum inward transport and endoplasmic reticulum protein degradation. Loss of function leads to salt sensitivity in plants [38]. The homologous gene AT2G41540 of Zm00001d042152 is related to glycerol metabolism andaffecting root development [39]. The homologous gene AT1G36580 of Zm00001d048723 encodes a 2,4-Dienoyl-CoA reductase related protein, involved in the metabolism of peroxisomes [40]. The homologous gene AT4G27090 of Zm00001d025857 encoded a protein that participates in the ribosomal large subunit biosynthesis process and is reported to be closely related to plant fertilization, which can positively regulate seed development [41]. The homologous gene AT1G07090 of Zm00001d038016 encodes the AtLSH6 protein, and its homologous protein AtLSH1 is functionally dependent on photosensitive pigments, which then mediates light regulation of seedling development [42]. AT1G18440, a homologous gene of Zm00001d038903, encodes a hydrodynamic protein and participates in transcription [43]. The homologous gene AT4G02930 of Zm00001d034856 encodes a Mitochondrial Elongation factor Tu, which can interact with Zn^2+^ and participate in the oxidative respiration function of mitochondria [44]. AT3G01550, a homologous gene of Zm00001d039674, encodes a phosphoenolpyruvate PEP/phosphate transporter AtPPT2, which plays an important role in chloroplasts and affects leaf development [45]. AT4G07960, a homologous gene of Zm00001d038676, encodes a protein of cellulose synthase like C12 (CSLC12), which is involved in the synthesis of xylan, consequently affecting, the formation of plant primary cell walls. It is necessary for the normal growth of root hairs and the interaction between pollen and pistil tissue [46]. The homologous genes AT4G29840 and AT1G72810 of Zm00001d038904 encode proteins AtMTO2 and AtTSY2 respectively, which are closely related to the development of plant root tip meristem [47]. We anticipate that the findings from this study will help determine the exact function of these genes in regulating the accumulation of trace element in maize and strengthen the planning of the implementation of MAS in maize breeding.

## Materials and methods

### Plant material and phenotyping

The association panel included 170 maize accessions from Dr. Lai’s laboratory at China Agricultural University [48]. These 170 accessions were planted at Hainan experimental station in SanYa city (18.75 N °, 109.17E °) in November 2013, serving as the first replicate (denoted E1) and Guangdong experimental station in Jiang Men city (22.61 N °, 113.06E °) in September 2020 and 2021 (denoted E2 and E3, respectively). At each location, the accessions were arranged in plots spaced 0.25 m within rows and 0.6 m between rows, following a randomized complete block design.

Well pollinated ears were harvested from each accession in the different environments. After manual threshing, representative mature grains from each accession were dried at 80℃ for 3 days and pulverized for measuring elements, including iron (Fe), manganese (Mn), molybdenum (Mo). Each sample weighing 5 g was digested in MARS6 microwave (CEM) with 65% nitric acid (superior purity) and covered and left for 1 h. Subsequently, the lid was screwed on the jar with a gradient of temperatures ranging from 120℃ to 180℃ for 45 min followed by a 30 min cooling period. The lid was slowly opened to vent the air, then the inner lid was rinsed with a small amount of water, and the digestion jar was placed on a temperature-controlled hot plate and heated at 100℃ for 30 min. After dilution with deionized water and fixed to 10 mL, the mixture was thoroughly mixed and set aside. A blank test sample was prepared the, samples were diluted in deionized water and the concentration of 3 elements (ug/g) were measured using inductively coupled plasma mass spectrometry (Agilent 7700 series). Finally, the mean of each element was calculated based on three replications.

### Statistical analysis of the elemental traits

The mean, range, standard deviation (SD), skewness, kurtosis, and coefficient of variation (CV) were calculated for the element concentration of the 170 maize accessions in different environments using the PYTHON software. The correlation analysis of each pair elements was performed using “corr” function, and we drew a heat-map correlogram. Analysis Of Variance (ANOVA) was performed to understand the interactions between genes and the environment (g×e) using the “Agricolae” package of R software. The Broad-sense heritability (h^2^) was calculated for elemental traits according Knapp [49] as: h^2^ = δ^2^_g_ / (δ^2^_g_ + δ^2^_e_ / E + δ^2^_g×e_ / RE ) where δ^2^_g_, δ^2^_e_, δ^2^_g×e_, E, and RE is genetic variance, residual variance, g×e variance, environment number, and product of the repeat number × the environment number, respectively.

### Genome-wide association mapping and LD analysis

A total of 170 accessions were performed genotyping-by-sequence (GBS) as described in our previous works [48]. The population structure was evaluated to obtain the Q matrix using Admixture (v1.3.0) with default parameters using 5-fold cross-validation, and hypothetical subgroup (K) values from 2 to 15, and finally we found That when K = 10, the maximum posterior probability value occurred, Therefore, the Q matrix was used for GWAS. Principal component analysis (PCA) was used to visualize the genetic relationships among samples using PLINK (v1.9), and10 PCs were used to better distinguish groups and fix the effect for GWAS. The Kinship matrix was calculated using EMMAX software with the parameters -v -h -s -d 10 [50].

A total of 3 million SNPs (3,123,762) with a minor allele frequency of ≥ 0.05 were used to perform GWAS via emmax software. Finally, using plink software with the parameters r ^2^ ≥ 0.2, window size = 50, and step size = 50, 116,011 independent SNPs were obtained.GWAS was performed to identify peak SNPs using the emmax software with Multiple Loci Linear Mixed (MLM_Q + Kinship and MLM_PCA + Kinship) Model using the mean of the phenotype. A threshold value of *P*-value < 0.5/116,011 = 4.31E-6 (Bonferroni correction) was used to detect significant SNPs.

Haploview 4.2 software [51] was used to perform the analysis of linkage disequilibrium (LD), and given the default parameters with allele spacing less than 500 kb and deletion rate less than 50%. Finally, LD analysis between alleles was performed using the subroutine “LD Plot”. The LD within 100 kb [52, 53] was used for searching candidate gene at the upstream or downstream.

### Superior allele analysis for ionomic element

The phenotypic effect value was calculated according to the method of [54]. The specific calculation formula is as follows: Ai=∑Nij/ni - ∑Ny/ny. Ai represents the phenotypic effect value of each QTN allele; Nij is the determination value of the jth material property phenotype carrying the ith QTN allele; ni is the number of materials with the ith QTN (i) allele; Ny representsthe phenotypic determination value of all materials; ny is the number of all materials. When Ai is < 0 or > 0, the QTN allele is considered as reduced (minus sign “-”) or synergistic (plus sign “+”) QTN allele. Here, given “+” mean the QTN allele is superior allele, while “-” as alternative allele. Then, ANOVA was performed evaluate the level of significance in the differences for phenotypic values of materials carrying different alleles using IBM SPSS Statistics 19 software.

### Candidate gene prediction, GO and KEGG Analysis

Potential candidate genes were searched on MaizeGDB (http://www.maizegdb.org) and NCBI (https://www.ncbi.nlm.nih.gov/), based on the peak SNPs. Next, a BLAST analysis (http://plants.ensembl.org/index.html) was performed for all identified candidate genes to search homologous genes and annotate the function compared with *Arabidopsis*. Gene Ontology (GO) and KEGG analysis was conducted to explain the functional categories using KOBAS v3.0 software (http://kobas.cbi.pku.edu.cn/).The analysis was performed using KOBAS v3.0, in which were observed significant enrichment of the zma00960 KEGG pathway, which plays a pivotal role in the biosynthesis of secondary metabolites in maize.

### Electronic supplementary material

Below is the link to the electronic supplementary material.

Supplementary Material 1

Supplementary Material 2

Supplementary Material 3

Supplementary Material 4

Supplementary Material 5

Supplementary Material 6

Supplementary Material 7

Supplementary Material 8

## Data Availability

As the data provider has not consented to the public sharing of the genotyping data for the maize inbred line, it cannot be shared on this platform. However, interested individuals can obtain the data by reaching out via email. To request access to the data, please contact the corresponding author, Dr. Xiangbo Zhang, at zhxiangbo@126.com.
